# Correspondence

**Published:** 1884-01

**Authors:** J. Lindsay

**Affiliations:** Huntington, L. I.


### CORRESPONDENCE

*To the Editor of the Journal of Comparative Medicine and Surgery:*

DEAR SIR—The October number of your journal contains an article by Dr. Carl Lemke, of Berlin, on hypodermic injections of morphine in the treatment of colic. The dose he recommends is from eight to ten grains, and he states that he has never found injurious results. I entirely agree with the doctor in his statement as I have myself given even larger doses and no injurious effects have ever followed, as, for instance:

In July, 1880, I was called to see a horse, fifteen hands high, ten years of age, which had been suffering during the previous forty-eight hours from impaction of the colon. I immediately injected hypodermically ten grains of morphine and four grains of atropine. The relief was instantaneous. I had him placed in a field so that should he have any pain during the night he would not injure himself. He lay down where I left him and rested all night. Was found grazing next morning and had had a movement of the bowels.

In October, 1883, I was called to see a horse that had been suffering for twelve hours and had taken six ounces of laudanum without obtaining relief. On my arrival I administered six grains of morphine hypodermically, and in ten minutes he lay down and slept.

I find that in all these cases hypodermic injections of morphine give relief in from ten to fifteen minutes, whether in recent attacks or in those of twelve hours standing. The dose I usually give is six grains. This treatment may be considered as a prognosticator, as the cases which it will not relieve are surely fatal.

As I have not seen any similar cases reported by the members of the profession, giving their experience in this treatment, I forward mine hoping they will be found worthy of occupying a space in your valuable journal.

Yours,

J. LINDSAY, D.V.S.

HUNTINGTON, L. I., Nov. 7th, 1883.

